# Accessibility, availability and utilisation of malaria interventions among women of reproductive age in Kilosa district in central Tanzania

**DOI:** 10.1186/1472-6963-14-452

**Published:** 2014-10-02

**Authors:** Susan F Rumisha, Maria M Zinga, Carolyn A Fahey, Dorothy Wei, Veneranda M Bwana, Malongo RS Mlozi, Elizabeth H Shayo, Robert C Malima, Benjamin K Mayala, Grades Stanley, Tabitha Mlacha, Leonard EG Mboera

**Affiliations:** National Institute for Medical Research, P.O. Box 9653, Dar es Salaam, Tanzania; Catholic University of Health and Allied Sciences- Bugando, Mwanza, Tanzania; Georgetown University, Washington, DC USA; Sokoine University of Agriculture, Chuo Kikuu, Morogoro, Tanzania

**Keywords:** Malaria, Pregnancy, Interventions, Mosquito nets, Preventive treatment, Tanzania

## Abstract

**Background:**

Universal access to and utilization of malaria prevention measures is defined as every person at malaria risk sleeping under a quality insecticide-treated mosquito net (ITN) and every pregnant woman at risk receiving at least two doses of sulfadoxine-pyrimethamine (SP). This study aimed to determine factors affecting accessibility, availability and utilisation of malaria interventions among women of reproductive age in Kilosa district in central Tanzania.

**Methods:**

Women of reproductive age with children <5 years old or those who had been pregnant during the past 5 years were included in the study. A structured questionnaire was used to seek information on malaria knowledge, accessibility and utilization of malaria interventions during pregnancy.

**Results:**

A total of 297 women (mean age=29±6.8 years) were involved. Seventy percent of the women had attained primary school education. About a quarter of women had two children of <5 years while over 58% had ≥3 children. Most (71.4%) women had medium general knowledge on malaria while only eight percent of them had good knowledge on malaria in pregnancy. A significant proportion of women were not aware of the reasons for taking SP during pregnancy (35%), timing for SP (18%), and the effect of malaria on pregnancy (45.8%). Timing for first dose of SP for intermittent preventive treatment in pregnancy (IPTp) was 1-3 months (28.4%) and 4-6 months (36.8%). Some 78.1% were provided with SP under supervision of the health provider. Knowledge on malaria in pregnancy had a significant association with levels of education (p*=*0.024). Ninety-eight percent had an ITN, mostly (87.1%) received free from the government. All women attended the ANC during their last pregnancy. The coverage of IPT1 was 53.5% and IPTp2 was 41.1%. The proportion of women making more ANC visits decreased with increasing parity.

**Conclusion:**

This study showed that the knowledge of the pregnant women on malaria in pregnancy and IPTp was average and is likely to have an impact on the low IPTp coverage. Campaigns that provide educational massages on the risk of malaria during pregnancy and the usefulness of IPTp need to be emphasised.

## Background

Malaria in pregnancy is one of the major public health problems in endemic areas of sub-Saharan Africa and has important consequences on birth outcomes [1]. Malaria infection during pregnancy can result in maternal death, maternal anaemia, low birth weight, pre-term birth and sometimes early infant death [2–8]. The current World Health Organization strategies to prevent and reduce malaria transmission in pregnant women include the use of intermittent preventive treatment in pregnancy (IPTp) and insecticide-treated nets (ITNs) [9]. Early diagnosis and proper case management compliment the preventive strategies.

The IPTp doses are delivered at health facilities during Antenatal Care (ANC) visits. Up to four doses can be provided, scheduled at each trimester and during delivery. Despite the high proportion of women who attend ANC services during their pregnancy, those who receive IPTp as per guidelines are still very few. In most cases only the first dose is well covered [9].

Tanzania adopted IPTp strategy as a national policy in 2001. According to nation-wide surveys, the proportion of women who received IPTp during ANC visits during their last pregnancy were 31% and 28%, in 2008 and 2010, respectively [10, 11]. Recent statistics indicate that the coverage of IPTp2 is still low at 31.3% [12]. IPTp2 coverage in Tanzania was among the highest in southern Africa in 2005 but it has dropped to third place after Zambia (63%) and Malawi (54%) in the most recent surveys [13]. The targets for the IPTp indicator in Tanzania were to have coverage of 60% by 2012 and 80% by 2015. On the other hand, dramatic progress has been made in the ownership and use of ITN in Tanzania. During 2008 to 2012 the proportion of pregnant women who slept under an ITN increased from 26% to 76% [10, 14]. The bulk of the increase occurred in rural areas where malaria transmission rates are highest. With targets set at 60% by 2010 and 80% by 2015 the trend is promising.

Universal access to and utilization of malaria prevention measures is defined as every person at malaria risk sleeping under a quality ITN and every pregnant woman at risk of malaria receiving at least two doses of SP administered during the second and third trimesters. Direct observation of SP by the health worker is highly recommended [15]. However, various factors including socio-economic status, urbanization and performance of health system influence the effectiveness of the strategy [16].

This study was carried out to determine factors affecting accessibility, availability and utilisation of malaria interventions among women of reproductive age in the rural community of Kilosa district in Tanzania. Specifically, the study aimed: (i) to determine the coverage of intermittent preventive treatment for malaria during pregnancy; (ii) to investigate the determinants of utilization of malaria preventive services among women who delivered in the previous five years; (iii) to assess health facility readiness in providing ANC services in relation to malaria in pregnancy; and (iv) to determine insecticide treated mosquito net coverage among women of reproductive age in the district.

## Methods

### Study area

The study was carried out in Kilosa District (22°17’-32°49’E and 9°127’-9°3339’N) in central Tanzania. The district has a total surface area of about 14,400 km^2^ and a population of 489,513 people living in 105,635 households with an average household size of 4.6 people. The climate belongs to the tropical savannah of the low latitude environment. The rainfall has a characteristic monomodal pattern; the rains begin in October with a peak in April and continue till May. The mean annual temperature is 25°C (mean annual maximum = 30°C; mean minimum = 19°C). Kilosa is malaria holoendemic area and studies have shown higher prevalence of low birth weight (17.5%) and stillbirth (4.8%) [17]. Kimamba Ward was purposively selected for the study due to the nature of the livelihoods of its community which include crop farming, nomadic pastoralism, mixed farming, petty business, and civil services. With these characteristics the population was considered appropriate to represent the district.

### Study design

This cross sectional quantitative community based survey was carried out during November 2011. The study involved all women of reproductive age (15–49 years) with biological children aged less than five years or those who had been pregnant in the past 5 years. The study subjects were women who were permanent residents in the selected ward of Kimamba. Purposive sampling was used to select women to participate in a study. The sample size was calculated assuming IPT2 coverage of 27% [11], a precision of 0.05 and 95% confidence interval. Using a standard formula for sample size calculation in cross sectional studies [18] and considering a dropout rate of 10% the sample sizes obtained was 332 (rounded to 330) individuals.

### Data collection

A structured questionnaire was used to interview women on malaria knowledge, availability, accessibility and utilization of malaria preventive measures. Social and demographic data including gender, marital status, education, occupation, distance to the closest health facility and costs related to accessing ANC services were collected. Women’ knowledge on the effect of malaria during pregnancy and utilization of SP for IPTp was also assessed. Key questions were: “When should a pregnant woman take the first dose of SP for intermittent preventive treatment of malaria?”; “What are the effects of malaria on the unborn baby?”; “How many times during the last pregnancy did you take SP tablets for IPTp at ANC clinic?” The antenatal cards were reviewed for those who attended ANC clinics to ascertain the information given by the respondents. Card information included gravidity, gestation age at first ANC visit, gestation age at first and second SP doses.

An assessment of the health facility readiness was done to identify healthcare service factors that influence the IPTp programme implementation. These included availability of SP and safe drinking water at the ANC; level of staff knowledge and training in IPTp; and supervision and monitoring of IPTp programme. A questionnaire was administered to staff of the selected ANCs in 10 health care facilities. In addition, non-participatory ANC observations were made using a standardized checklist.

### Data management and analysis

The database was prepared using EpiData Version 3.1 software and data were entered using trained clerks. The data cleaning and quality check was done by a statistician by comparing 10% of the questionnaires with the entered data. Data was migrated to STATA (Stata Corps) for further analysis. Cross tabulations were done between selected variables of interest. Association between different factors was assessed using *χ*^2^-test and proportional test was used to test difference in proportions between sub-groups.

**Figure 1:**
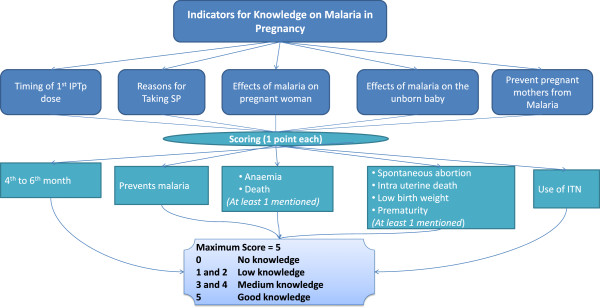
**Assessment of women’ knowledge on malaria in pregnancy in Kilosa.**

The key outcome variable was defined using responses from the question “*how many times during the last pregnancy did you swallow SP tablets at ANC?*” This binary response variable categorized those who received the recommended dosage of SP and those who did not. The categorization was done in a way that those who responded once were coded = 0 and those mentioned twice/ thrice were coded = 1. The information was counterchecked by reviewing ANC cards. The knowledge on malaria in pregnancy was assessed based on the response of women on the five key indicators. These were knowledge on timing of first dose of SP (IPT1), reasons for taking SP, effects of malaria on the mother, effect on unborn baby, and malaria prevention methods. A correct response on each of these was given one score making a maximum of 5 points. These were later categorized into 4 groups range - from “0 = no” to “5 = good” knowledge (Figure 1). A binary variable categorizing 0, 1 and 2 *versus* 3, 4 and 5 was later established.

Two separate multivariate logistic regression models were fitted to identify effect of individual characteristics such as age, occupation or parity, on the knowledge of malaria in pregnancy and SP utilization. The model for SP utilization was adjusted for distance to the nearest healthcare facility with ANC service (which was defined by time taken to reach the facility), frequency of ANC visits during last pregnancy, trimester when the first visit was made, average time spent on a single ANC visit and knowledge of malaria in pregnancy. For health care workers, the level of knowledge on IPTp, training on IPTp in the past 12 months, availability of potable water and SP at the health facility, directly observed treatment (DOT) practice for SP and monitoring and supervision of IPTp in ANCs were analysed.

### Ethical considerations

This study received ethical approval from the Tanzania Medical Research Coordinating Committee of the National Institute for Medical Research. Permission to conduct the study was sought from the Kilosa District Council, Ward and Village authorities. Meetings convened by Ward and Village Executive Officers were used to explain the objective of the study. The Ward and Village Executive Officers were also used during the recruitment of the study subjects. Verbal Informed consent was obtained from each respondent before the interview. The consent of each subject was recorded on the survey form.

## Results

### Demographic characteristics

A total of 297 women (15–49 years) participated in this study. The mean age of the respondents was 29 years (SD = 6.8). Three quarters of the women (76.4%) were married or living with partners. Almost all women (92%) were involved in crop farming as the main source of income. About 70% of the women had at least a primary school education (Table 1).

**Table 1:** **Socio-demographic characteristics in women of reproductive age in Kilosa**

Variable	Response	N (%)
**Age category (years)**	15-19	25 (8.4)
	20-24	54 (18.2)
	25-29	79 (26.6)
	30+	139 (46.8)
Education	At least primary school	207 (69.7)
	None	90 (30.3)
Marital Status	Married/living together	227 (76.4)
	Others (single, divorced/separated, widowed)	70 (23.6)
Status in the household	Head	36 (12.1)
	Spouse	224 (75.4)
	Daughter	37 (12.5)
Occupation	Crop production	273 (91.9)
	Others (Business, Employed)	2 (8.1)
Number of Children	One	51 (17.2)
	Two	76 (25.6)
	> Three	170 (57.2)
Number of children < 5 yrs	One	227 (76.4)
	Two	61 (20.5)
	Three	7 (2.4)
	Four	2 (0.7)
Total		297

Proportion of educated women increased with age, with those aged 30 years or older accounting for the largest proportion (43%, p = 0.019, Cuzick’s test for trend). About one fifth of the women had two children under the age of five at the time of the survey, and 3% (n = 9) had three or more children (Table 1). About 2% of the women aged >45 years old had an underfive child. There was a significant association between the number of underfives and education status. Those with at least a primary school education had a lower proportion of having more than three underfive children (Two-sample test of proportions, p *=*0.023).

### Knowledge on malaria in pregnancy

**Figure 2:**
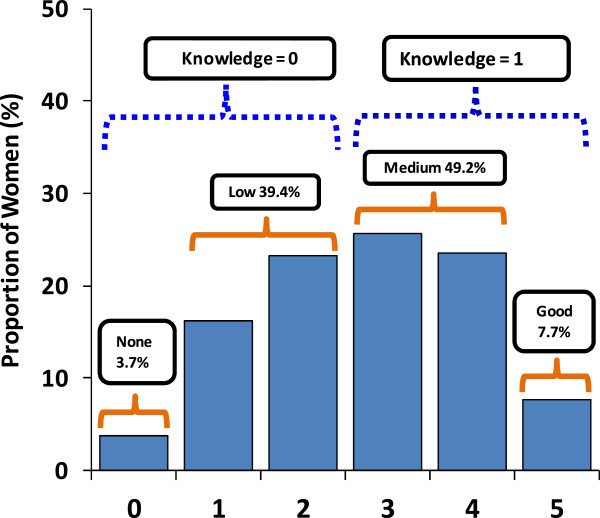
**Categorization of knowledge on malaria in pregnancy scores obtained from women of reproductive age, Kilosa.**

The assessment indicated that about 4% of the women could not show any indication of having knowledge on malaria in pregnancy despite the fact that all women mentioned to attend antenatal care services at least once and claimed to receive information on malaria prevention and treatment. Only 7.7% of the women had good knowledge on malaria in pregnancy. The rest of the respondents were classified as having medium or low knowledge levels (Figure 2).

**Figure 3:**
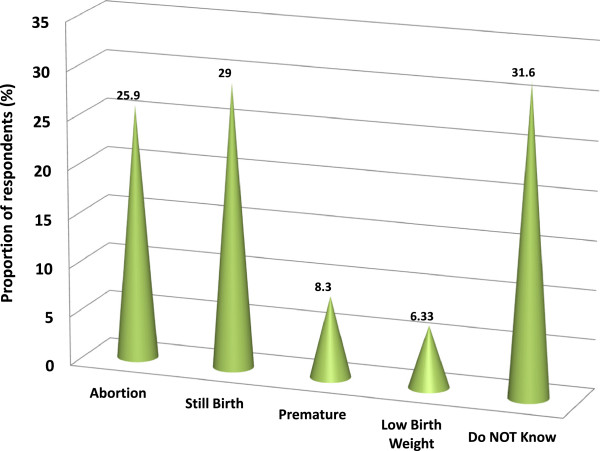
**Knowledge on the effect of malaria to the unborn baby in Kilosa.**

Close to half (44.1%) of the women did not know the reasons for taking of SP while over half (58.4%) did not know the right time for taking SP for IPT (at least the first dose). In addition, over half (58.9%) of the respondents were not aware of effect of malaria on a pregnant woman. Knowledge on the effect of malaria to the unborn baby among mothers was generally poor (Figure 3). Results of the regression model (Table 2) depicts significant association between knowledge of malaria in pregnancy and level of education (OR*:* Educated *=* 1.7, 95% CI: 1.1, 2.8) and woman’s age (OR: 20–24 years =2.3 vs. 30+ years = 4.3). Other variables including having more underfives, type of occupation or marital status had no significant association with good knowledge of malaria in pregnancy.

**Table 2:** **Results of the regression model assessing the effect of different predictors on knowledge on malaria in pregnancy and receiving two doses of SP as malaria preventive strategy during pregnancy**

Response	Predictor	OR (95% CI)	P-value
**Knowledge on malaria in pregnancy**	**Age category (years)**		
	20-24	2.3 (0.8, 6.6)	0.124
	25-29	3.3 (1.2, 8.9)	0.02*
	30+	4.3 (1.6, 11.7)	0.004*
	**Education**		
	Educated (>Primary School)	1.7 (1.1, 2.8)	0.046*
	**Status in the household**		
	Spouse	2 (1.0, 4.2)	0.06
	Daughter	1.8 (0.6, 5)	0.263
**Receiving two doses of SP**	**Age category (years)**		
	20-24	2.0 (0.5, 7.5)	0.302
	25-29	3.0 (0.9, 10.5)	0.077
	30+	3.3 (1.0, 10.8)	0.05*
	**Time spent at ANC clinic**		
	1-2 hr	0.7 (0.4, 1.4)	0.349
	3-4 hours	0.7 (0.3, 1.5)	0.38
	5-6 hours	0.2 (0.1, 0.8)	0.016*

**Key**: OR = Odd ration; CI = Confidence Interval; SP = sulfadoxine-pyrimethamine; ANC = antenatal care; *Significant factors.

### Access and utilization of malaria interventions

Over 97% of the women mentioned to have consulted a health facility when suffering from malaria. Health care was mostly sought from the public facilities (98%). Alternative sources of care included traditional healers and medical drug stores or use of leftover medicines at home. These options were described to be due to high cost related to accessing the conventional facilities and low satisfaction with quality of service at health facility.

Almost all (98%) women had at least a mosquito net in their household. The average number of mosquito nets per household was 2.6 (SD = 1.3). Among those with mosquito nets, 87.1% received them free the from the government distribution programmes. Other sources of nets were through the national voucher scheme (18%) and self purchase. About 12% of the women had mosquito nets obtained from both free distribution and voucher schemes. The attitude towards effectiveness of mosquito nets in preventing malaria did not differ between those who received free nets and those who self purchased the nets (p > 0.05). Most women (96.2%) mentioned to use mosquito nets to prevent themselves from malaria. Cleaning of the environment (21%) and draining of stagnant water (15%) were the other methods of malaria prevention mentioned by the respondents.

All women reported attending antenatal care from health facilities during their last pregnancy. Proportionally, 44.7% attended three times and 42.7% over 3 times. A small proportion (12.5%) attended two times or less. There was a positive association between the number of attendance and age of the woman. The first visit was commonly during the first trimester, mostly (60.6%) during the third month of gestation. However, a significant proportion (39.4%) of women attended the ANC clinic during the second or third trimesters. The proportion of ANC attendance during the first and second month of gestation was 0.35% and 1.4%, respectively. Interestingly, 6.7% of the respondents attended the ANC after the 6^th^ month of gestation.

**Figure 4:**
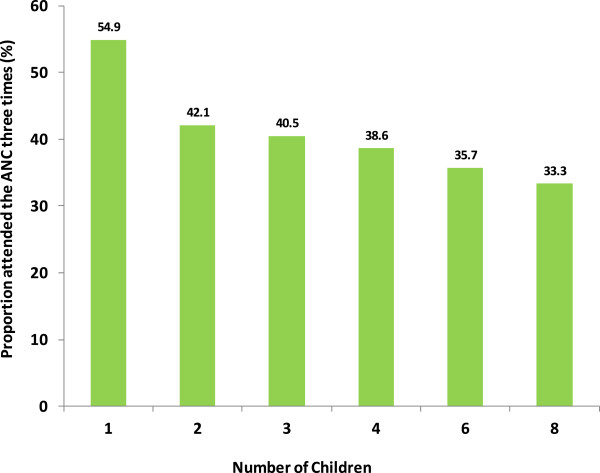
**Relation between number of children and frequency of attending scheduled ANC services in Kilosa.**

The number of children of the respondent was related to the number of ANC visits. A larger proportion of women who made three or more ANC visits were those with only one child. The proportion of ANC visits declined with increasing parity (Figure 4). Timing for first dose of SP for IPTp was mentioned to be 1–3 months (28.4%) and 4–6 months (36.8%). Over three quarters of women (78.1%) took SP under supervision of a health care provider. Assessing the coverage of IPTp1 and IPTp2 it was observed that, over half (53.5%) claimed to receive SP tablets during ANC visit once during the last pregnancy. Some 41.1% reported to receive the recommended doses of SP. The responses were confirmed through examination of the ANC cards by the investigators.

Respondents with high knowledge of malaria had more ANC visits than those with low knowledge of malaria in pregnancy. Three quarters (75%) of women with low knowledge attended ANC only once compared to 28% of those with high knowledge. On the other hand, 62% of the women with high knowledge and 42% of those with low knowledge made more ANC visits.

**Figure 5:**
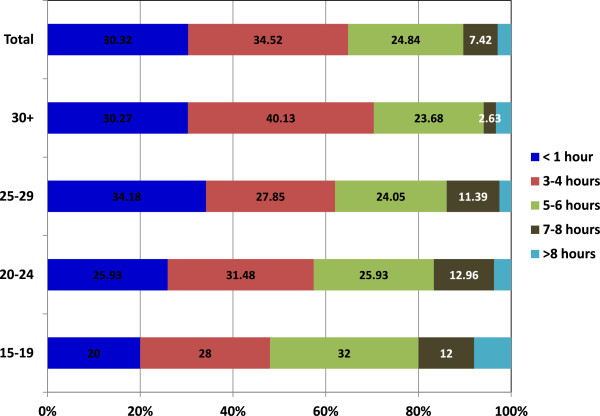
**Average time spent for ANC visit including travel, waiting and service time in Kilosa.**

The distance to the nearest health facility for each respondent was assessed. It was observed that facilities that provide ANC were close and accessible by the majority of the respondents. Over half (56.8%, n = 176) of the respondents walked for 15–30 minutes to the health care facility. One quarter used less than 15 minutes and the other used more than 30 minutes. On average, 30% of women spent less than 1 hour during their ANC visit (including travel, waiting and service time). Some 34.5% of the respondents reported to spend between 3–4 hours for travel, waiting and service time for any single ANC visit. Younger women reported to spend longer times than the older ones (Figure 5).

**Figure 6:**
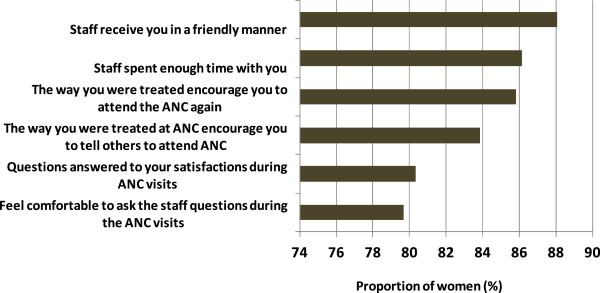
**Levels (in %) of the satisfaction of women on the antenatal care service in Kilosa.**

Over 30% of the women claimed to suffer from malaria during their last pregnancy and 95% of them were treated at a health facility. The antimalarial drugs used for treatment were either SP (44.4%), Artemether-Lumefantrine (28.7%) or quinine (11.1%). Three quarters (75%) of the women who attended ANC at least once reported having delivered at health facility. Women were satisfied with the quality of ANC services provided at the facilities (Figure 6).

From a regression model it was observed that individual factors significantly related with receiving two doses of SP were age (OR = 3.3 for women >30 years) and time spent in the clinic which was negatively related reducing the chance for about 80% (OR = 0.2, 95% CI: 0.1, 0.8). Factors such as number of children, education, distance to health facility, number of ANC visits, gestation period at first visit of ANC, frequency of attendance, knowledge of the effect of malaria on pregnancy and usefulness of SP to the mother/unborn child were not statistically significant (Table 2).

### Health facility readiness

Ten facilities were visited and 26 health workers interviewed. The facilities included three health centres, seven dispensaries and one district hospital. Of the total of 26 ANC staff, 6 were nurse-midwives, 5 nurses, 8 medical attendants, 6 clinical officers and 1 assistant medical officer. Only three health facilities offered laboratory malaria diagnosis. Only the ANC clinic at the district hospital had a capacity to provide blood transfusion services (Table 3). Twenty-five (96%) of the health workers interviewed admitted to experience stock outs of SP for a period from 1 to 12 months during the period of one year preceding the study. Stock out of SP was reported in 90% of the visited facilities. During the period of stock outs, the ANC staff were either suspending IPTp services or advising their clients to come back on the next visit to receive SP. Alternatively, the health workers would issue prescription for the women to buy SP elsewhere. Potable water for taking SP was available at all the ANC clinics visited.

**Table 3:** **Characteristics of health facilities in Kilosa**

Type of HF	Name of health facility	No. of staff	IPTp-SP outreach services	SP stock out	Laboratory present	Provision of blood transfusion services
Hospital	Kilosa RCH	11	No	1 month	Yes	Yes
Health Centre	Kimamba	4	No	6 months	Yes	No
	Masange	5	Yes	No	Yes	No
	Ulaya	11	Yes	8 months	No	No
Dispensary	Rudewa	4	Yes	6 months	No	No
	Kisanga	4	Yes	1 month	No	No
	Msimba	2	No	12 months	No	No
	Mvumi	5	Yes	8 months	No	No
	Ilonga	5	Yes	12 months	No	No
	Chanzuru	3	Yes	6 months	No	No

All health workers interviewed had good knowledge on IPTp, although the majority of them did not know the contraindications of SP. Of the 26 health workers interviewed only one had formal training on IPTp during the 12 months preceding the study. All ANCs had the IPTp national guidelines. Forms for adverse events were not available in all ANCs. Direct observed treatment (DOT) of SP was practiced in all ANCs visited. Surprisingly, none of the health workers admitted to having received a supervision visit specific for the IPTp programme during the 12 month period before the study.

## Discussion

The IPTp-SP programme has not been successfully implemented in Kilosa district as the coverage is below the national target of 60% for IPT2. SP stock out and lack of qualified health workers was common in all health facilities in the district. Despite high awareness, positive attitudes, and appropriate treatment seeking behaviour among women, completion of the SP regimen was still low with only 41.3% of the women completing two doses of SP as recommended by the World Health Organization [19]. A recent analytical review of the Health Sector Strategic Plan III of Tanzania has indicated that the national coverage of two doses of SP during antenatal visits is still low despite a slight increase from 29.6% in 2008 to 31.3% in 2012 [12, 14]. The poorest wealth quintiles have the lowest IPT2 coverage (25%) than women in the best-off quintile, which is also still below 50%.

Factors such as long waiting and service times at the health facilities for the ANC service are likely to play a role in the observed patterns. Studies elsewhere in Tanzania and sub-Saharan Africa have indicated a combination of lack of awareness, health worker behaviour, stock outs and policy as possible explanations for low IPTp coverage rates [20–22]. In Zambia, where high coverage of IPTp second dose has been achieved and sustained within existing systems, highlights coordinated support to the routine clinic system and training to antenatal care workers as key enabling factors [23]. A study in north-east Tanzania reported that health facility workers do aim to stay within the recommendations for IPTp delivery, and reported missed opportunities for protecting pregnant women with two doses [22]. Tanzania antenatal guidelines differ from the current WHO recommendations which require provision of SP to all pregnant women at their first ANC visit after quickening [19, 24]. This is designed to be easily implemented and stays within safety limits of SP in pregnancy; and is not dependent on individual timing preferences for first attendance to antenatal clinic. In a recent meta-analysis, key barriers to the provision of IPTp and ITNs were listed to include unclear policy and guidance on IPTp; general healthcare system issues, such as stock-outs and user fees; health facility issues stemming from poor organisation, leading to poor quality of care; poor healthcare provider performance, including confusion over the timing of each IPTp dose; and women's poor antenatal attendance, affecting IPTp uptake [25].

In this study, there was no pattern on attendance with the age of the woman. However, the parity was observed to be related to the number of ANC visits; with the proportion of ANC visits declining with increasing parity. Moreover, respondents with high knowledge of malaria had more ANC visits than those with low knowledge of malaria in pregnancy. Similar to our findings, a study in southern Tanzania showed age was not associated with second SP dose coverage [26]. In a recent study in Nigeria, older age bracket, ever attended school, currently living with a partner, ever married, and wealth were significantly associated with compliance with taking of SP for IPTp [27]. Marital status, education level and occupation are expected to be predictive of receiving more doses of SP but this was not observed in this study. The free maternal health care policy could have helped overcome these factors as barriers to accessing healthcare through the ANCs. Other studies have also shown that marital status, educational level and household socio-economic status are not associated with a second dose of SP [26, 28].

This study showed that approximately two thirds of the respondents attended ANC for the first time when they were in their first trimester and a third of them during their second trimester. In a study in Kenya it was observed that about two thirds of women first visited the ANC in the third trimester [29]. The late attendance was associated with perceived lack of quality in the ANC services. Despite the early ANC attendance observed in our study, only 41.3% of the respondents received two doses of SP – indicating that early attendance does not contribute to the increase in coverage of the second SP dose. Late first ANC registration has been found to contribute to incomplete IPTp [16, 29, 30].

In this study, readiness of the health facilities to provide the ANC services could be one of the major factors contributing to the poor IPTp coverage. Although potable water for DOT was available free of charge in almost all the health facilities in the district, SP stock out was common and frequent. Frequent SP stock out are likely to contribute to low coverage of IPTp in the district. Interruptions in supply of SP to the health facilities have the potential of negatively influencing the IPTp programme [16]. This potentially undermines the increased ANC attendance by the pregnant women that the free maternal health care policy seeks to achieve if medicines to be given are not available. Unavailability of potable water in Malawi and drug stock-outs in Kenya, Uganda and Zambia have been described to impact IPTp coverage levels [31]. To ensure DOT is being practiced as recommended in the guidelines for IPTp, availability of SP and potable water as well as commitment of the health workers to observe all pregnant women take SP at ANC is needed.

Compliance to IPTp guidelines, quality and content of the health education given at the ANC clinic depends on the knowledge and training of the ANC staff on IPTp. The findings from this study revealed that although only one health worker had formal training on IPTp in the previous year, the level of knowledge of the staff on malaria in pregnancy was average. Unfortunately, the staffs were not aware of the availability of IPT guidelines in their respective facilities. A study in Kenya has demonstrated an increase in IPTp coverage after health care workers were re-trained on IPTp [32]. Formal training and retraining of the health workers is an important factor in improving coverage of malaria and other health interventions. Moreover, regular provision of supportive supervision of the frontline health workers including aspects related to delivery of ANC services has been shown to be strong motivational factors. Usually, each health facility in Tanzania has to have supervision at least once every three months by the members of district Council Health Management Team. However, findings from this study showed this doesn’t happen regularly. Similar to our findings, in a study in northern Tanzania, health workers complained that their supervision was not systematic and not supportive when provided [33]. Several studies have shown supportive supervision is essential for quality improvement and job satisfaction [34, 35]. However, supervisors themselves are often poorly resourced and may not be trained in effective supervision techniques. This is an important area for development in improving the Tanzanian health service.

This study is likely to have faced some limitations. The respondents may not have recollected all that happened during their ANC visits during their last pregnancy leading to recall bias. However, this was minimized by examining ANC records and compared to the answers given by the respondents and this was found to be comparable.

## Conclusion

In conclusion, this study showed high coverage of insecticide treated nets but low coverage of IPTp among pregnant women in Kilosa district. The knowledge of the pregnant women on IPTp was average and could have impacted on IPTp coverage. The implementation of simplified IPT guidelines would be critical for reaching the 80% national target for IPTp by 2015. Campaigns that provide educational massages on the risk of malaria during pregnancy and the usefulness of IPTp so as to raise clients’ awareness should be emphasised. The government must make sure that SP is available in all public facilities at all times. Improving upon the monitoring and supervision of the programme as well as ensuring regular training and re-training of health workers in IPTp will further improve the programme in the district and the country at large.

## References

[1] De Beaudrap P, Turyakira E, White LJ, Nabasumba C, Tumwebaze B, Muehlenbaachs A, Guérin PJ, Boum Y, McGready R, Piola P (2013). Impact of malaria during pregnancy on pregnancy outcomes in a Ugandan prospective cohort with intensive malaria screening and prompt treatment. Malaria J.

[2] Teplin SW, Burchinal M, Johnson-Martin N, Humphry RA, Kraybill EN (1991). Neurodevelopmental, health, and growth status at age 6 years of children with birth weights less than 1001 grams. J Pediat.

[3] Steketee RW, Wirima JJ, Hightower AW, Slutsker L, Heymann DL, Breman JG (1996). The effect of malaria and malaria prevention in pregnancy and on offspring birthweight, prematurity, and intrauterine growth retardation in rural Malawi. Am J Trop Medicine Hyg.

[4] Steketee RW, Nahlen BL, Parise ME, Menendez C (2001). The burden of malaria in pregnancy in malaria-endemic areas. Am J Trop Med Hyg.

[5] Taylor HG, Klein N, Minich NM, Hack M (2000). Middle-school-age outcomes in children with very low birth-weight. Child Develop.

[6] Guyatt HL, Snow RW (2001). Malaria in pregnancy as an indirect cause of infant mortality in sub-Saharan Africa. Trans Royal Soc Trop Med Hyg.

[7] Guyatt HL, Snow RW (2004). Impact of malaria during pregnancy on low birth weight in Sub-Saharan Africa. Clin Microb Rev.

[8] Grantham-McGregor S, Cheung YB, Cueto S, Glewwe P, Richter L, Strupp B (2007). Developmental potential in the first 5 years for children in developing countries. Lancet.

[9] WHO (2012). World Malaria Report.

[10] TDHS (2005). Tanzania Demographic and Health Survey, 2004–2005.

[11] TDHS (2011). Tanzania Demographic and Health Survey 2010.

[12] THMIS (2013). Tanzania HIV/AIDS and Malaria Indicator Survey 2011–12.

[13] MoHSW/IHI/NIMR/WHO (2013). Midterm Analytical Review of the Performance of the Health Sector Strategic Plan III 2009-2015.

[14] THMIS (2008). Tanzania HIV/AIDS and Malaria Indicator Survey 2007-08.

[15] Mutagonda R, Kamuhabwa AAR, Massawe S, Mpembeni R (2012). Intermittent Preventive Therapy and Treatment of Malaria during Pregnancy: A Study of Knowledge among Pregnant Women in Rufiji District, Southern Tanzania. Trop J Pharm Res.

[16] Mubyazi GM, Magnussen P, Goodman C, Bygbjerg IC, Kitua AY, Olsen OE, Byskov J, Hansen KS, Bloch P (2008). Implementing intermittent preventive treatment for malaria in pregnancy: review of prospects, achievements, challenges and agenda for research. Open Trop Med J.

[17] Uddenfeldt Wort U, Hastings I, Mutabingwa TK, Brabin BJ (2006). The impact of endemic and epidemic malaria on the risk of stillbirth in two areas of Tanzania with different malaria transmission patterns. Malaria J.

[18] Lwanga SK, Lemeshow S (1991). Sample Size Determination in Health Studies: A Practical Manual.

[19] WHO: *Intermittent Preventive Treatment of malaria in pregnancy using Sulfadoxine-pyrimethamine (IPTp-SP)*. Updated WHO Policy Recommendation (October 2012); http://www.who.int/malaria/iptp_sp_updated_policy_recommendation_en_102012.pdf

[20] Mubyazi G, Bloch P, Kamugisha M, Kitua A, Ijumba J (2005). Intermittent preventive treatment of malaria during pregnancy: a qualitative study of knowledge, attitudes and practices of district health managers, antenatal care staff and pregnant women in Korogwe District, North-Eastern Tanzania. Malaria J.

[21] Tarimo DS (2007). Appraisal on the prevalence of malaria and anaemia in pregnancy and factors influencing uptake of intermittent preventive therapy with sulfadoxine-pyrimethamine in Kibaha District, Tanzania. E Afr J Pub Hlth.

[22] Anders K, Marchant T, Chambo P, Mapunda P, Reyburn H (2008). Timing of intermittent preventive treatment for malaria during pregnancy and the implications of current policy on early uptake in north-east Tanzania. Malaria J.

[23] Steketee RW, Sipilanyambe N, Chimumbwa J, Banda JJ, Mohamed A, Miller J, Basu S, Miti SK, Campbell CC (2008). National Malaria Control and Scaling Up for Impact: The Zambia Experience through 2006. Am J Trop Med Hyg.

[24] WHO (2007). Malaria in Pregnancy. Guidelines for Measuring Key Monitoring and Evaluation Indicators.

[25] Hill J, Hoyt J, van Eijk AM, D’Mello-Guyett L, ter Kuile FO, Steketee R, Smith H, Webster J (2013). Factors affecting the delivery, access, and use of interventions to prevent malaria in pregnancy in Sub-Saharan Africa: A systematic review and meta-analysis. PLoS Med.

[26] Marchant T, Nathan R, Jones C, Mponda H, Bruce J, Sedekia Y, Schellenberg J, Mshinda H, Hanson K (2008). Individual, facility, and policy level influences on national coverage estimates for intermittent preventive treatment of malaria in pregnancy in Tanzania. Malaria J.

[27] Onyeneho NG, Orji BC, Okeinbunor JC, Brieger WR (2013). Characteristics of Nigerian women taking sulfadoxine/pyrimethamine twice during pregnancy for the prevention of malaria. Int J Gyn Obst.

[28] Mbonye AK, Neema S, Magnussen P (2006). Preventing malaria in pregnancy: a study of perceptions and policy implications in Mukono District, Uganda. Hlth Policy Plan.

[29] van Eijk AM, Bles HM, Odhiambo F, Ayisi JG, Blokland IE, Rosen DH, Adazu K, Slutsker L, Lindblade KA (2006). Use of antenatal services and delivery care among women in rural western Kenya: a community based survey. Reprod Hlth.

[30] Ndyomugyenyi R, Katamanywa J (2010). Intermittent preventive treatment of malaria in pregnancy (IPTp): do frequent antenatal care visits ensure access and compliance to IPTp in Uganda rural communities?. Trans Roy Soc Trop Med Hyg.

[31] Hill J, Kazembe R (2006). Reaching the Abuja target for intermittent preventive treatment of malaria in pregnancy in African women: a review of progress and operational challenges. Trop Med Int Health.

[32] Ouma PO, van Eijk AM, Hamel MJ, Sikuku E, Odhiambo F, Munguti K, Ayisi JG, Kager PA, Slutsker L (2007). The effect of health care worker training on the use of intermittent preventive treatment for malaria in pregnancy in rural western Kenya. Trop Med Int Hlth.

[33] Manongi RN, Marchant TC, Bygbjerg IC (2006). Improving motivation among primary health care workers in Tanzania: a health worker perspective. Hum Res Hlth.

[34] Ahmed AM, Desta A, Tekle K, Mweta EA (1993). Pursuing better health care delivery at district level. World Health Forum.

[35] Ben Salem B, Beattie KJ (1996). Facilitate supervision: a vital link in quality of reproductive health service delivery. Engender Health Working Paper No. 10.

[CR36] The pre-publication history for this paper can be accessed here: http://www.biomedcentral.com/1472-6963/14/452/prepub

